# RNA therapeutics

**DOI:** 10.1080/15476286.2022.2161704

**Published:** 2023-01-11

**Authors:** Tony Gutschner

**Affiliations:** Junior Research Group ‘RNA Biology and Pathogenesis’, Faculty of Medicine, Martin Luther University Halle-Wittenberg, Halle, Germany

RNA is a versatile molecule with unique properties that allows it to fulfil many different tasks within a living cell. Besides its important role as a messenger between the DNA and protein worlds, RNA can adopt many different secondary and tertiary structures which allow also non-coding RNAs to engage in various cellular processes by serving as scaffolds to assemble protein complexes or acting as sponges that sequester RNAs or proteins [1]. These multifunctional properties of RNA as well as its ubiquitous distribution within cells and organisms have attracted much attention recently, especially in the field of medicine. Several initiatives have been launched to explore and leverage the therapeutic potential of RNA. One of the most impressive examples, both in terms of speed and efficacy, was the development of a mRNA vaccine for use in combating the coronavirus disease 2019 (COVID-19) [2]. However, unique challenges remain during the drug discovery and manufacturing process, which need to be overcome in order to implement RNA therapeutics and leverage their full potential.

This special issue on *RNA therapeutics* is comprised of a selection of original research articles and expert reviews and aims to provide a comprehensive overview about the different types of RNA-based therapeutics and their mode of action. Halloy et al. introduce two established categories of nucleic acid therapeutics, antisense oligonucleotides (ASOs) and small interfering RNAs (siRNAs) as well as emerging RNA-based technologies, with a focus on their potential as biosensors and therapeutics [3]. In addition, Karen Anthony provides a comprehensive overview about additional RNA-based drug modalities such as RNA aptamers, RNA-based vaccines and mRNA drugs and highlights their current and potential applications, especially in the field of neurological diseases [4]. Furthermore, Malard et al. and Xiaolin Wang et al. focus on the important process of RNA splicing and its regulation, e.g. by non-coding RNAs, and introduce therapeutic concepts to modulate splicing reactions by ASOs and small-molecule splicing switches [5,6]. Since mRNA vaccines have gained much attention recently, Rouf et al. delineate the evolution of the technology and explore the immense potential it offers as a prophylactic option for other diseases besides COVID-19 [7]. Another technology which holds great clinical potential is Clustered Regularly Interspaced Short Palindromic Repeats (CRISPR)-based genome editing. Mechanistically, the CRISPR system relies on a guide RNA (gRNA) and an RNA-guided Cas nuclease. The gRNA can be easily manufactured and assembled with Cas proteins into a ribonucleoprotein (RNP) complex *in vitro* for subsequent delivery into cells to induce gene knockouts or site-specific mutations using additional repair templates. In order to efficiently generate point mutations and simplify the delivery process, Ghosh et al. developed chimeric oligonucleotides encoding a target-specific gRNA fused to a single-stranded DNA repair template for CRISPR RNP-mediated precision genome editing [8].

While these articles showcase the broad versatility of RNA and RNA-targeting drugs, there are also challenges associated with RNA therapeutics. One obstacle lies in the recognition of unmodified RNA molecules in mammalian cells by pattern recognition receptors, which function in the detection of molecular patterns associated with pathogens or cellular damage. A subset of these pattern recognition receptors is activated by the atypical presence and/or location of double-stranded RNA or its synthetic analogue polyinosinic-polycytidylic acid, also referred to poly(I:C). This can trigger a pro-inflammatory signalling and cell death response, and Sales Conniff et al. show that RNA sensing effects, especially the effect on cell viability, can be amplified by electroporation of poly(I:C) in murine cancer cells [9]. Hence, the delivery of poly(I:C) and its derivatives by electroporation could be tested as single-agent regimen or in combination with other anti-cancer modalities in the future. However, in order to achieve a broad usability of RNA therapeutics, in particular for protein expression applications like in the case of mRNA vaccines, chemical modification of RNA is critical to prevent non-specific cell toxicity [7]. Moreover, chemical modifications are also critical to stabilize RNAs or achieve a tissue- and cell-type specific delivery. In articles by Holm et al. and Gangopadhyay et al., several modification strategies are reviewed and delivery strategies are discussed with a special focus on ASO drugs and siRNA-based therapeutics [10,11]. Yet, despite major improvements and recent successes, development of RNA therapeutics is still limited by inefficient delivery of oligonucleotides to specific tissues and organs. Bakowski and Vogel address these and other challenges of RNA therapeutics and summarize the evolution of complexity of oligonucleotide delivery methods with a special focus on increasing complexity of formulations from gymnotic delivery to bioconjugates and to lipid nanoparticles [12]. Another challenge, that is not solely limited to RNA therapeutics, is the translation of *in vitro* results into experimental models *in vivo* as well as species-specific constraints and effects that complicate the development and testing of efficient drugs. This is highlighted in a research study presented by Kilikevicius et al. in which the authors were not able to achieve increased frataxin (FXN) expression using anti-FXN oligonucleotides in a mouse model of Friedreich's ataxia besides the positive effects previously observed in patient-derived fibroblasts as well as in induced pluripotent stem cell-derived neuronal progenitor cells [13]. Therefore, the development of RNA therapeutics and their pre-clinical testing requires a comprehensive understanding of the disease and needs to consider evolutionary pitfalls like differences in nucleotide sequences and conservation of relevant effector or target molecules, especially when working with non-coding RNAs.

Next to their use as direct molecular targets or effector molecules, RNAs can be of diagnostic value and can be leveraged as sensitive biomarkers of different diseases. Along these lines, Kakouri et al. present the first genome-wide next-generation small RNA sequencing in serum samples of five different types of muscular dystrophy patients and healthy individuals and identified many small RNAs including miRNAs, lncRNAs, tRNAs, snoRNAs and snRNAs that differentially discriminate the muscular dystrophy patients from the healthy individuals [14]. In another biomarker study, Eyileten et al. identified and validated microRNAs with a role in ACE2-related thrombosis in coronavirus infection, which could serve as novel, thrombosis-related predictive biomarkers for COVID-19 complications and could be used for early patient stratification [15]. Besides altered RNA expression levels, one should also consider the alternative splicing as well as chemical modification and editing of RNA as exemplified in studies by Shizhi Wang et al., Amweg et al. and Xu et al. [16–18]. This will allow us to take full advantage of the diagnostic and prognostic potential of coding and non-coding RNAs in the future.

In conclusion, the articles presented herein highlight the tremendous potential as well as the challenges of RNA therapeutics. Further research is needed in order to engineer and deliver patient-specific RNA-based drugs. One aspect that should be addressed in the future is the intimate connection between RNA and its associated proteins. Drugs that interfere with this interaction hold great potential as exemplified in a study by Wallis et al. [19]. Furthermore, natural products and secondary metabolites like polyphenols should be tested and their potential effects on RNA expression and processing should be investigated in order to gain a deeper understanding of their mode of action as suggested by Shirazi-Tehrani et al. [20].
